# Rethinking Accessibility in Light of the Orange Declaration: Applying a Socio-Ecological Lens to Rural Mental Health Commissioning

**DOI:** 10.3389/fpsyt.2022.930188

**Published:** 2022-06-24

**Authors:** Mathew Coleman, Beatriz Cuesta-Briand, Noel Collins

**Affiliations:** ^1^Rural Clinical School of WA, The University of Western Australia, Crawley, WA, Australia; ^2^Telethon Kids Institute, Perth Children's Hospital, Nedlands, WA, Australia; ^3^Western Australia Country Health Service (WACHS), Albany, WA, Australia

**Keywords:** orange declaration, commissioning, mental health, rural, accessibility

## Abstract

The prevalence of mental illness is a critical public health issue. In Australia, the prevalence of mental illness is similar across all settings, however, people living in rural and remote areas experience worse outcomes than their urban counterparts. Access to mental health services is critical, however, the notion of accessibility needs to be understood in the context of the uniqueness and variability of the rural experience. The *Orange Declaration on Rural and Remote Mental Health* recognized that rural areas face a series of interconnected challenges and called for place-specific responses and new funding models that reward collaboration and local partnerships. In this paper, we argue that recent mental health planning, policy and service development uses a narrow interpretation of the notion of accessibility that is out of step with current thinking on the heterogeneity of the rural experience. We use some examples of our own research and experience in rural Western Australia to argue that the current commissioning model is not aligned with the Orange Declaration, and remains largely metro-centric and reliant on a narrow conceptualization of service accessibility. We argue that what is needed is a dynamic, responsive, context-sensitive understanding of accessibility that is informed by the distinctiveness of rural adversity, and recognizes the heterogeneity and variability of the rural experience whilst acknowledging rural agency and social capital, and we suggest that applying a socio-ecological approach to the development of new commissioning models provides a way forward.

## Introduction

The prevalence of mental illness is a critical public health issue, both in Australia (1) and internationally (2). The negative impact of the social distancing and lockdown measures implemented throughout the COVID-19 pandemic on mental health has brought the issue of mental wellbeing to the forefront of the public debate, and Governments must urgently respond to the challenge of providing adequate mental health services to meet an increasing demand.

In Australia, the prevalence of mental illness is similar across all settings, however, people living in rural and remote areas experience worse outcomes than their urban counterparts (3). Of particular concern is the incidence of suicide, with monitoring data from the Australian Institute for Health and Welfare for the year 2020 showing significantly higher age-standardized rates for regional and rural areas compared with major cities – in very remote areas, the rate is more than double (22.9 per 100,000 compared with 10.3) (4).

Mental health service gaps have been identified in rural areas (5), and a 2018 Senate Inquiry Report recognized access to quality mental health care in rural areas as a pressing national issue (6). Despite the Fifth *National Mental Health and Suicide Prevention Plan* prioritizing the importance of ensuring that all Australians with a mental illness can access effective and appropriate treatment and community support (7), accessibility remains a pivotal issue in rural mental health, but one that must be understood in the context of the uniqueness and variability of the rural experience.

There is growing evidence of the impact of adversity on mental health outcomes of people living in rural and remote areas (8). Released in 2019, the *Orange Declaration on Rural and Remote Mental Health* (the Orange Declaration) recognized that rural areas face a series of interconnected challenges, including geographical, demographic, social, economic, technological and environmental, which are not adequately addressed by current mental health service models (9). The Orange Declaration emphasized contextual variance (8) and called for place-specific responses and new funding models that reward collaboration and local partnerships (9).

In this paper, we argue that recent mental health planning, policy and service development uses a narrow interpretation of the notion of accessibility that is out of step with current thinking on the heterogeneity of the rural experience. If mental health outcomes for people living in rural and remote areas are to be improved, we argue that the notion of accessibility requires reframing as a response to the Orange Declaration and in the context of the current understandings of the distinct characteristics of “rural adversity”. We propose that a more dynamic, context-sensitive model of accessibility should be adopted as the basis for mental health service commissioning within rural community contexts.

## Accessibility as Geographical Availability: the 2020 Productivity Commission's Report

Access to services is a critical issue in mental health service delivery and is one of the most widely used concepts in discussing care systems. Faced with the evidence on the inequality of rural mental health outcomes, and in the context of an increased public awareness of the societal and economic burden of mental illness and suicide, it is tempting (and, we argue, simplistic) for policy makers to regard “more” – more services, more training, more telehealth – as the solution to the issue of service accessibility. Whilst there is no question that rural service gaps exist that require an expansion in mental health services and the mental health professional workforce, evidence shows that there are other issues at play such as the under-utilization and inefficiencies of existing services (10). If policy makers want to develop effective strategies to tackle accessibility issues in rural areas, it is critical that they look beyond whether services purely exist or not. Access to health services can be conceptualized as having five domains: approachability (users can identify that services exist); acceptability (factors determining whether or not users will accept the services); availability and accommodation (health services can be reached both physically and in a timely manner); affordability (economic capacity to use the services); and appropriateness (quality, adequacy, coordination and continuity of care) (11). Working on Penchansky and Thomas' original theory of access (12), Saurman proposed a sixth domain, awareness, which the author conceptualized as having a dual dimension: awareness about a service on the users' side, and awareness of local context on the services' side (11, 13). Service availability (as indicated by geographical service location) is sometimes used as a proxy for accessibility; in the rural context, this is overly simplistic and problematic.

The much-awaited Productivity Commission's Mental Health Inquiry (PCMHI) report into the Australian mental health system was released in November 2020, presenting a long-term plan to improve efficiencies in mental health services in Australia. The report followed a 2-year inquiry and resulted in a number of system-wide priority reform recommendations. These included: prevention and early help; improving people's experience with mental healthcare; improving people's experiences with services beyond the health system; and instilling incentives and accountability for improved outcomes (14). Disappointingly, place-based solutions as proposed by the Orange Declaration to meet the needs of rural and remote communities were largely overlooked, in favor of high-tech solutions such as remote video-conferencing and workforce incentivisation (14). Geographical location (availability) and scale (quantity) appear to be the main measures adopted by the PCMHI report with respect to service provision, with scant recognition of the heterogeneity and variability associated with rural circumstances and rural adversity across Australian communities outside large metropolitan centers.

Universally, the PCMHI recommendations would likely benefit individual- and community-level mental health and wellbeing if implemented. The economic benefits of the recommendations were estimated to be between $1.3–$18 billion per year as a result of the increased economic participation of people with mental ill-health (14). The distribution of these potential benefits is not outlined in the report, and it is reasonable to assume that it would mirror the aforementioned disproportionate disadvantage experienced by rural and remote communities.

A promising recommendation for rural Australia from the report was the preferred “rebuild model” approach to funding and commissioning arrangements. Cooperative regional funding pools administered by Regional Commissioning Authorities (RCAs) are recommended to overcome “unnecessary and inefficient discontinuities, duplication and gaps” that exist between funding providers, levels of government and service purchasers (14). Regional decision-making through joint regional planning and governance oversight are consistent with the place-based, integrated and locally codesigned services and systems called for in the Orange Declaration. However, this promising recommendation has not received universal support, perhaps owing to the lack of granularity in coordinating commissioning models, processes, evaluation and learning systems within the RCA model. This is in contrast to a growing policy shift toward population approaches and devolved commissioning models that has occurred internationally, of which local commissioning groups in the United Kingdom are an example (15).

Jurisdictional attempts to consider rural and remote commissioning have been undertaken in Australia in the past, with variable success. In 2016, the Queensland Mental Health Commission published its *Queensland Rural and Remote Mental Health and Wellbeing Action Plan 2016-18* (16). Predictably, the plan set out with laudable aims to: reduce the incidence, severity and duration of mental illness and mental health problems; reduce suicide and its impact; and prevent and reduce the adverse impact of alcohol and other drugs, in rural and remote communities. Specific and lofty goals were set out under three priority areas: better opportunities for good mental health and wellbeing; community strength; and responsive and accessible services. Evaluation of the action plan outcomes has been limited to date. In 2017, a ‘Shared Commitment’ dashboard indicated that only 1 of 28 actions had been completed, with no follow-up or explanatory data available since (16).

Poor coordination, collaboration and integration, and competing priorities between local [e.g. Primary Health Networks (PHNs)], state (e.g. jurisdiction Mental Health Commissions) and federal (e.g. Commonwealth Department of Health) commissioning bodies contribute to the lack of progress toward effective regional commissioning efforts (14, 17). The result is continued disparity in mental health outcomes for smaller, more heterogenous communities typically found in rural and remote areas.

Despite the almost universal goals set out in various state and national reports and plans, the following questions remain: how can regional commissioning address accessibility issues to materially improve the mental health and wellbeing of rural and remote communities? And what is required to ensure a dynamic, adaptive and iterative approach to the commissioning of services in the context of rural diversity that accommodates variation within the experience of rural adversity over time? Our own research and experience in alcohol and other drug (AOD), subacute mental health services and older adult psychiatry in rural Western Australia (WA) provide some clues.

## Context-Based Accessibility: the Orange Declaration

In our recent research on substance users' experiences of accessing AOD services in WA's South West region, we applied a socio-ecological lens and identified service gaps and limited intra- and inter-sectoral collaboration, most notably between community-based state-funded services and primary healthcare providers and between the AOD and the mental health services sectors (18). We also found that existing local services were able to effectively respond to a crisis such as the COVID-19 pandemic by assertively and flexibly adapting their service provision to suit local needs, against urban-centric commissioning parameters (19).

Our results showed that, whilst some service gaps can only be addressed by increasing availability (e.g. detoxification units are not available in the region and participants reported having to travel to Perth) and optimizing the distribution of services within regions, much would be gained by addressing other access issues, namely: approachability (e.g. knowledge of and information on available services), acceptability (e.g. service trustworthiness), accommodation (e.g. outreach and assertive service delivery), affordability (e.g. travel expenses and consultation fees), and appropriateness (e.g. continuity of care and coordination between services). Our results also suggested that more granular, context-specific rural service planning would be more effective at harnessing local social capital.

Furthermore, we recently undertook an evaluation of a newly established residential sub-acute mental health community service in the Great Southern region (unpublished data, manuscript under review), which demonstrated a number of similar issues relating to accessibility, including: approachability (e.g. barriers within referral pathways and assessment); acceptability (e.g. cultural safety for Aboriginal clients); accommodation (e.g. insufficient reach to culturally and linguistically diverse community); and appropriateness (e.g. inconsistent coordination between services and lack of funding for post-discharge follow-up). Many of these problems appeared to relate to “cookie cutter” state commissioning and assumptions that metro-centric governance models would be fit for purpose in a rural context, rather than developing more nuanced, context-sensitive, local models.

Similarly, current state-wide commissioning and service models do not appear to be consistently meeting the mental health care needs of older Western Australians living in rural and remote regions. As there are no specialist beds outside of Perth, older people with complex psychiatric problems in rural settings are either treated in general adult psychiatric or acute medical beds, or require aeromedical retrieval to Perth, away from family and local supports, and straining Royal Flying Doctor Service capacity (20). There is also considerable variation across non-metropolitan regions in access to defined Older Adult Mental Health (OAMH) community services and more reliance on generic “ageless services” where clinical outcomes are known to be poorer (21). This variation in accessibility is due to a number of factors. There has been a relative policy vacuum, with the latest state-level OAMH strategy being published in 1998 (22). Since then, the absolute number of people over 65 have doubled (23) and demand for services has increased. Many rural clinicians feel disempowered to challenge an array of metropolitan misconceptions made by centralized commissioning bodies about the needs of their local communities (9). As a result, it is difficult for local clinicians to broker local solutions if the commissioning and policy focus remains fixed on narrow measures of accessibility such as state-wide bed capacity and flow. Regional commissioning, through PHNs, has not been able to address unmet needs as most of its funding has been limited, inflexible and targeted at youth and early intervention (24).

Whilst most regions in WA lack the economy of scale to provide specialist older adult inpatient care, regional capacity could be increased through a range of responses informed by the Orange Declaration, the principles of localism, and more dynamic and adaptive devolved commissioning. Whilst is it known that the latter is not a panacea to improve outcomes (24), more flexible models of care, tailored to both local clinical needs and realities, can be achieved through improved connectivity between existing providers, clinicians and communities (25). However, this needs to be supported by a clear policy framework and engagement with consumers and their carers (26) to connect “policy, people, and place” (9). An example in one WA regional center has been the implementation of a stepped care approach to make appropriate use of the existing repertoire of care settings for older people requiring intensive treatment. Non-frail older adults requiring inpatient care are managed in an ageless acute psychiatric unit, whilst frail individuals are treated through shared care arrangements with local GPs (in residential aged care facilities or local hospital settings) or with a local geriatrician in a hospital subacute unit. Only individuals with complex needs who cannot be treated locally by these arrangements are referred for metropolitan specialist admission. This approach has increased local capacity, facilitated care close to home and reduced the need to transfer to Perth, and was achieved through mobilizing existing social capital and strong working relationships (27). Another service improvement project, which evaluated local memory assessment pathways, revealed that there was inadequate post diagnostic support and highlighted the need for a dementia navigator (28). Whilst both state and regional commissioning bodies showed little interest in this finding, funding for the position was brokered through conversations with local stakeholders.

In contrast with a metro-centric, top-down commissioning approach, a psychiatry support line for General Practitioners (GPs) has been in operation since early last year in WA's Great Southern region, providing an innovative solution to improving access to mental health care. Developed and commissioned by the local PHN agents, the service addresses accessibility issues in a manner which is more consistent with access theory (11–13). Rather than focusing on service availability as measured by the psychiatry specialist workforce, and the number of subacute and inpatient beds, the psychiatrist-led support line is improving access to adequate mental health service by increasing GPs' capacity to manage their patients' mental health problems within the primary care setting. The service addresses approachability through exacting local knowledge from regionally based psychiatrists and GPs, builds on trust between service providers and, thus, acceptability for GPs and their patients, and accommodates a demand for immediacy by being available by phone in a timely, if not instant, manner. Furthermore, the service enhances affordability for users, both GPs and patients, and appropriately addresses coordination of care between providers in a manner consistent with in reach of specialist services into primary care. Importantly, the rural patient is at the center of care delivery.

The support line is currently being evaluated, and preliminary results suggest that the service has the potential to benefit all stakeholders (patients, primary health care providers, psychiatry specialists and the taxpayer) by allowing people with a mental illness to receive adequate care in the primary practice setting and consequently easing the referral pipeline.

The Orange Declaration calls for new service models tailored to context and better-aligned funding models that reward collaboration (9). Our experience and recent research on AOD and mental health services provide evidence that the current commissioning models are not aligned with the Orange Declaration, and remain largely metro-centric and reliant on a narrow conceptualization of service accessibility that does not take into account the heterogeneity of the rural experience (18, 19).

Insofar as it provides insights into the specificity of the rural experience of access to mental health services, access theory can be seen as congruent with and complementing the Orange Declaration. A nuanced, multi-layered conceptualization of access and accessibility issues underpinned by a socio-ecological approach is helpful at exploring local contexts and providing the required granularity for service development and delivery. We suggest that such an approach would be more likely to result in new commissioning models for rural areas that: 1) reward polycentric service planning, streamlined referrals and multi-sectorial collaboration; 2) acknowledges rural agency; and 3) harness the local social capital (existing ecosystems of support).

## Conclusion

We have argued that the current commissioning model reliant on a static notion of 'accessibility as availability' (geographical location) is flawed. What is needed is a dynamic, responsive, context-sensitive understanding of accessibility that is informed by the distinctiveness of rural adversity, and recognizes the heterogeneity and variability of the rural experience whilst acknowledging rural agency and social capital. This will aid the development of high quality mental health services for all Australians no matter where they live.

## Data Availability Statement

The original contributions presented in the study are included in the article/supplementary material, further inquiries can be directed to the corresponding author/s.

## Author Contributions

All authors listed have made a substantial, direct, and intellectual contribution to the work and approved it for publication.

## Funding

Funds received for academic purposes from University of Western Australia.

## Conflict of Interest

The authors declare that the research was conducted in the absence of any commercial or financial relationships that could be construed as a potential conflict of interest.

## Publisher's Note

All claims expressed in this article are solely those of the authors and do not necessarily represent those of their affiliated organizations, or those of the publisher, the editors and the reviewers. Any product that may be evaluated in this article, or claim that may be made by its manufacturer, is not guaranteed or endorsed by the publisher.

## References

[1] Australian Institute of Health and Welfare. Australia's Health 2020: In brief. Australia's health series no. 17 Cat. no. AUS 232. Canberra: AIHW. (2020).

[2] World Health Organization. Mental health (2022). Available online at: https://www.who.int/health-topics/mental-health (accessed June 13, 2022).

[3] ABS. National Survey of Mental Health and Wellbeing: Summary of Results. Canberra: Australian Bureau of Statistics; (2008).

[4] Australian Institute of Health and Welfare. Deaths by suicide by remoteness area (2021). Available online at: https://www.aihw.gov.au/suicide-self-harm-monitoring/data/geography/suicide-by-remoteness-areas (accessed June 13, 2022).

[5] van Spijker B Salinas-Perez J Mendoza J Bell T Bagheri N Furst M. Service availability and capacity in rural mental health in Australia: Analyzing gaps using and Integrated Mental Health Atlas. australian & New Zealand. J Psychiat. (2019) 53:1000–12. 10.1177/0004867419857809 31250654

[6] The Senate: Community Affairs References Committee. Accessibility and quality of mental health services in rural and remote Australia. Senate Inquiry Report. Canberra: Commonwealth of Australia. (2018).

[7] Department of Health. The Fifth National Mental Health and Suicide Prevention Plan. Canberra: Commonwealth of Australia. (2017).

[8] Lawrence-Bourne J Dalton H Perkins D Farmer J Luscombe G Oelke N. What is rural adversity, how does it affect wellbeing and what are the implications for action? Int J Environ Res Public Health. (2020) 17:7205. 10.3390/ijerph17197205 33019735 PMC7578975

[9] Perkins D Farmer J Salvador-Carulla L Dalton H Luscombe G. The Orange Declaration on rural and remote mental health. Australian J Rural Health. (2019) 27:374–9. 10.1111/ajr.12560 31515882

[10] Fitzpatrick SJ Handley T Powell N Read D Inder KJ Perkins D. Suicide in rural Australia: A retrospective study of mental health problems, health-seeking and service utilisation. PLoS ONE. (2021) 16:e0245271. 10.1371/journal.pone.0245271 34288909 PMC8294514

[11] Levesque J-F Harris MF Russell G. Patient-centred access to health care: conceptualising access at the interface of health systems and populations. Int J Equity Health. (2013) 12:18. 10.1186/1475-9276-12-18 23496984 PMC3610159

[12] Penchansky R Thomas JW. The concept of access: Definition and relationship to consumer satisfaction. Med Care. (1981) 19:127–40. 10.1097/00005650-198102000-00001 7206846

[13] Saurman E. Improving access: modifying penchansky and thomas' theory of access. J Health Serv Res Policy. (2016) 21:36–9. 10.1177/1355819615600001 26377728

[14] Productivity Commission. Mental Health - Report no. 95. Canberra. (2021).

[15] McDermott I Checkland K Coleman A Osipovič D Petsoulas C Perkins N. Engaging GPs in commissioning: realist evaluation of the early experiences of Clinical Commissioning Groups in the English NHS. J Health Serv Res Policy. (2017) 22:4–11. 10.1177/1355819616648352 27151153 PMC5207294

[16] Queensland Mental Health Commission. Queensland Rural and Remote Mental Health and Wellbeing Action Plan 2016-18. Brisband: Queensland Mental Health Commission. (2016).

[17] Ham C Timmins N. Managing health services through devolved governance: A perspective from Victoria, Australia. (2015). Available online at: https://www.kingsfund.org.uk/publications/managing-health-services-devolved-governance-victoria-australia (accessed June 13, 2022).

[18] Cuesta-Briand B Taran M Coleman M. A rural ecosystem of recovery: Lessons from substance users' experiences of accessing services in Western Australia's South West. Drug Alcohol Rev. (2022) 41:963–73. 10.1111/dar.13455 35315552

[19] Coleman M Taran M Cuesta-Briand B. Responding to rural adversity: a qualitative study of alcohol and other drug service users' experiences of service response to COVID-19 in Western Australia's Southwest. Australasian Psychiatry. (2022) 30:74–8. 10.1177/10398562211036125 34496219 PMC8894904

[20] Gardiner F Collins N Coleman M Quinlan F. Lack of dementia services may contribute to retrievals. Australian J Dement Care. (2021) 10:35–7. Available online at: https://journalofdementiacare.com/about-ajdc/

[21] Warner J. Wither old age psychiatry? Int Psychoger. (2014) 26:1055–8. 10.1017/S1041610214000544 24713475

[22] Health Department of Western Australia. Policy and Strategic Directions for Mental Health Services for Older People. Perth: Health Department of Western Australia. (1998).

[23] Australian Institute of Health and Welfare. Older Australians - Demographic profile 2021 Available online at: https://www.aihw.gov.au/reports/older-people/older-australians/contents/demographic-profile#Geographical%20distribution (accessed June 13, 2022).

[24] McGorry PD Hamilton MP. Stepwise expansion of evidence-based care is needed for mental health reform. Med J Austral. (2016) 204:351–3. 10.5694/mja16.00120 27169969

[25] Briggs DS Nankervis R Baillie J Turner C Rigby K Livingstone L. Innovations to improve patient care in Australian Primary Health Network: An insider's perspective. Public Admin Policy. (2019) 22:111–24. 10.1108/PAP-09-2019-0017

[26] Henderson J Javanparast S McKean T Freeman T Baum F Ziersch A. Commissioning and equity in primary care in Australia: Views from Primary Health Networks. Health Soc Care Commun. (2017) 26:80–9. 10.1111/hsc.12464 28608451

[27] Collins N. Older Adult Mental Health in the WA Country Health Service: Internal Report. (2022).

[28] Collins N. Great Southern Memory Clinic Proposal Report: Older Adult Mental Health. Albany, Western Australia: WA Country Health Service. (2020).

